# Evidence of chikungunya virus infections among febrile patients at three secondary health facilities in the Ashanti and the Bono Regions of Ghana

**DOI:** 10.1371/journal.pntd.0009735

**Published:** 2021-08-30

**Authors:** Jonathan Akwasi Adusei, Patrick Williams Narkwa, Michael Owusu, Seth Agyei Domfeh, Mahmood Alhassan, Emmanuel Appau, Alimatu Salam, Mohamed Mutocheluh

**Affiliations:** 1 Department of Clinical Microbiology, School of Medicine and Dentistry, Kwame Nkrumah University of Science and Technology, KNUST Main Campus, Kumasi, Ghana; 2 Department of Medical Diagnostics, Faculty of Allied Health Sciences, Kwame Nkrumah University of Science and Technology, KNUST Main Campus, Kumasi, Ghana; 3 Manhyia District Hospital, Manhyia, Kumasi, Ghana; WRAIR, UNITED STATES

## Abstract

**Background:**

Chikungunya is now of public health concern globally due to its re-emergence in endemic areas and introduction into new areas of the world. Worldwide, the vectors for transmission of the chikungunya virus are *Aedes* mosquitoes and these are prevalent in Ghana. Despite its global significance, the true burden of chikungunya virus infection in Ghana is largely unknown and the threat of outbreak remains high owing to international travel. This study sought to determine chikungunya virus infection among febrile patients suspected of having malaria infections at some selected health facilities in the Ashanti, Bono East, and Bono Regions of Ghana.

**Methodology:**

This cross-sectional study recruited six hundred (600) febrile patients suspected of having malaria who submitted their clinical samples to the laboratories of the selected health facilities for the diagnosis of their infections. Five to ten millilitres (5-10ml) of venous blood were collected from each study participant. Sera were separated and tested for anti-chikungunya (IgM and IgG) antibodies using InBios ELISA kit following the manufacturer’s instruction. Samples positive for chikungunya IgM and IgG were selected and tested for chikungunya virus RNA using Reverse Transcription-quantitative Polymerase Chain Reaction. Malaria Rapid Diagnostic Test kits were used to screen the participants for malaria. Structured questionnaires were administered to obtain demographic and clinical information of the study participants.

**Result:**

Of the 600 samples tested, the overall seroprevalence of chikungunya was 6%. The seroprevalence of chikungunya IgM and IgG antibodies were 1.8% and 4.2% respectively. None of the chikungunya IgM and IgG positive samples tested positive for chikungunya RNA by RT-qPCR. Of the 600 samples, tested 32.3% (194/600) were positive for malaria parasites. Malaria and chikungunya co-infection was detected in 1.8% (11/600) of the participants.

**Conclusion:**

Findings from the current study indicate low-level exposure to the chikungunya virus suggesting the virus is circulating and potentially causing morbidity in Ghana.

## Author summary

Chikungunya fever is a mosquito-borne disease of Chikungunya virus (CHIKV) origin that shares similar symptoms with malaria fever. The symptoms include fever, joint pains, muscle pains, skin rashes and headache. There is a paucity of information about CHIKV epidemiology in Ghana although the mosquito vector (*Aedes*) responsible for the transmission is present in the country. Ghana’s neighbours (Ivory Coast and Burkina Faso) have also reported isolated cases of CHIKV infections. The authors sought to find evidence of the circulation of CHIKV among suspected malaria patients in Ghana and to shed light on the importance of the investigation of non-malaria causes of fever such as chikungunya fever. And the possible inclusion of chikungunya fever in the differential diagnoses of unexplained febrile cases in Ghana. We found out that 6% of the 600 people studied had antibodies to the CHIKV i.e. they were previously infected with CHIKV. Malaria prevalence was 32.3% among the study participants. The rate of malaria-chikungunya co-infections was 1.8% among the study participants. Our findings show a low-level exposure to the CHIKV suggesting the virus is circulating and potentially causing morbidity in Ghana that may be misdiagnosed as malaria.

## Introduction

Chikungunya virus (CHIKV) which was first reported in Tanzania in 1952 is a spherical, enveloped virus with a diameter of about 60–70 nm [1–3]. The virus belongs to the genus Alphavirus in the Togaviridae family. The virus has a linear single-stranded, positive-sense RNA genome of approximately 12 kb. The viral genome has two open reading frames (ORFs) with the first ORF encoding for four non-structural proteins (nsp1, nsp2, nsp3, and nsp4) while the second ORF encodes for five structural proteins (C, E3, E2, 6K, E1) [4,5]. The glycoproteins E1 and E2 play a key role in the replication of the virus; thus, while glycoprotein E1 is involved in membrane fusion, glycoprotein E2 facilitates the entry of the virus into the host cell through endocytosis [6]. Phylogenetic studies have identified four different genotypes of CHIKV that have been implicated in epidemics. These genotypes are the West African, Asian, East-Central-South African (ECSA), and Indian Ocean Lineage (IOL) [7,8]. The CHIKV causes chikungunya fever, an acute febrile illness characterized by severe joint pain, muscle pain, joint swelling, headache, nausea, fatigue, and skin rash. CHIKV infections are almost always self-limiting and rarely fatal [9]. The main long-term complication associated with CHIKV infections is the persistence of joint pain and stiffness and this may last for years after the initial infection has resolved [10]. The primary vectors implicated in the transmission of CHIKV are infected mosquitoes of the *Aedes* species predominantly *Aedes aegypti* and *Aedes albopictus* [11,12]. Two studies have shown that *Aedes aegypti* mosquitoes are actively circulating in Ghana and could potentially promote outbreaks of viral haemorrhagic fevers [13,14].

In Africa, sporadic and epidemic cases of CHIKV infections have been reported in several countries including Cameroon, Senegal, Central African Republic, South Africa, Angola, Nigeria, Burkina Faso, Ivory Coast, Benin, Guinea [15]. The disease has now spread to other continents and cases have been reported in countries in the Americas, Europe, and Asia [16]. International travel, transmission efficiency, and global distribution of the vectors have facilitated the spread of the virus into new geographic regions [17].

Even though CHIKV infections have not been officially reported in Ghana, autochthonous cases or outbreaks have been reported in Ghana’s neighbouring countries such as Burkina Faso, Ivory Coast, Benin, and Nigeria in recent years [18,19]. This coupled with the presence of an active vector population in Ghana, international travel, and trade among West African countries increases the risk of an outbreak in the nearest future. Chikungunya and other Aedes-borne febrile infections share similar symptoms with malaria. Furthermore, the fact that most health facilities in Ghana lack adequate diagnostic tools for Aedes-borne viruses often leads to misdiagnosis of these infections as malaria [20]. The misdiagnoses of many of these Aedes-borne febrile illnesses as malaria often threaten public health and significantly contribute to the high cost of health care in Ghana. It is therefore important that we document the exposure levels of CHIKV among febrile patients in Ghana. The current study sought to determine CHIKV exposure among febrile patients in some selected health facilities in the Ashanti, Bono East, and Bono Regions of Ghana. The outcome of the study would provide baseline data on which measures could be instituted to prevent outbreaks of CHIKV infection in Ghana.

## Materials and methods

### Ethics statement

Ethical approval was sought from the Committee on Human Research, Publications, and Ethics of School of Medicine and Dentistry (SMD), Kwame Nkrumah University of Science and Technology (KNUST), Kumasi-Ghana (Approval letter number: CHRPE/AP/479/19). Permission was also sought from the Institutional Review Board (IRB) of the selected health facilities. Formal written consent was obtained from individual participants after explaining the purpose of the study in a language each participant understood.

### Study design, sample size, and study site

This hospital-based cross-sectional study was conducted at the Holy Family Hospital, Techiman, Bono East Region; Sunyani Municipal Hospital, Sunyani, Bono Region; and the Manhyia District Hospital, Kumasi, Ashanti Region between September 2018 and November 2019.

The sample size was estimated based on the seroprevalence of 41.8% of chikungunya virus infections in two states of Southwest Nigeria reported by Olajiga *et al*., 2017 [19]. The detailed calculation is illustrated below:

$$n=\frac{Z^{2}\times P(1-P)}{M^{2}}=\frac{1.96^{2}\times 0.418(1-0.418)}{0.05^{2}}=373.82$$

*Where*:

*n* = sample size; Z = z-score or standard score value from the standard normal distribution reflecting the confidence level (at confidence level 95%, the z-score = 1.96); M = margin of error of 5.0%; P = estimated seroprevalence of 41.8%.

The estimated sample size was 374, which implied the minimum number of participants to be recruited.

The study recruited 661 febrile patients who submitted their clinical samples to the laboratories of the selected health facilities for laboratory diagnosis of malaria. However, 61 (9.22%) of the samples taken haemolysed and were not suitable for the serological test so were excluded from the study. Therefore, the total number of subjects recruited for the study was 600 and the breakdown was 213, 227 and 221 from Manhyia Hospital, Kumasi, Sunyani Municipal Hospital, Sunyani and Holy Family Hospital, Techiman, respectively.

### Eligibility criteria

Inclusion criteria for enrolment included patients suspected of having malaria with the onset of symptoms not exceeding 15 days and having one or more of the following symptoms: arthralgia, myalgia, fever, and other malaria-like symptoms including headache, nausea, vomiting, chills, and shivering.

### Data and blood sample collection

Structured questionnaires were administered to obtain demographic information of the study participants. Five to ten millilitres (5–10 ml) of whole blood samples were collected from each participant by venipuncture into serum separation tubes. The samples were allowed to clot and then centrifuged at 1500 rpm for 5 minutes to separate the sera. The serum samples were aliquoted into two separate vials of 1 ml and temporarily stored at -20°C at the laboratories in the selected health facilities before being transported to the Virus Research Laboratory at the Department of Clinical Microbiology, SMD-KNUST where the samples were stored frozen at -20°C until testing.

### Malaria diagnosis

Malaria Rapid Diagnostic Test kits (CareStart, USA) were used to screen the participants for malaria. Each cassette was labelled with the patient’s serial number. Five microliters (5 μl) of the blood sample was placed into the sample (S) pad. Two drops of the sample diluent assay (buffer) were dispensed into the diluent pad and allowed to run. The results were interpreted within 20 minutes. Lines on both the control (C) and test (T) indicated a positive result, which signified the presence of histidine-rich protein 2 (HRP 2) of *Plasmodium falciparum* indicative of the presence of the parasites in the patient. A line on only the ‘C’ indicated a negative result. No line at all or on the ‘C’, indicated an invalid result, and the test was performed again.

### Serological detection of anti-chikungunya IgM and IgG

Serological detection of anti-chikungunya IgM and IgG were done using CHIKjj Detect IgM and CHIKjj Detect IgG ELISA kits (InBios International Inc. Seattle, USA) following instructions and recommendations from the manufacturer. The reagents and the samples were allowed to equilibrate at room temperature before the assay was performed. CHIKV IgM and IgG positive and negative controls as well as the serum samples were first diluted with the sample dilution buffer (1:100). Fifty microliters (50 μl) of the diluted controls and samples were added to the designated wells of the micro-titre plates and then incubated at 37°C for 30 minutes. After washing, 50 μl of CHIKV antigen was added to each well and incubated at 37°C for 30 minutes. Washing was repeated after which each well (except for the blank) was treated with 50 μl of enzyme conjugate for CHIKV and then incubated at 37°C for 30 minutes. After subsequent washing, substrate solution 3,3’,5,5’-tetramethylbenzidine (TMB) hydrogen peroxide was added to each well (including the blank well) and incubated in the dark at room temperature (20–25°C) for 10 minutes. A stop solution was added to each well and incubated at room temperature for 1 minute after which the optical density (OD) of each well was measured at 450 nm using iMark Microplate Reader (Bio-Rad, USA). The ELISA results were interpreted following the instructions of the manufacturer of the test kits used. The sensitivity and specificity of the test kits were greater than 90% according to the manufacturer.

### Reverse transcription-polymerase chain reaction (RT-qPCR)

Serum samples that tested positive for CHIKV IgM and IgG were selected and further tested using RT-qPCR. CHIKV RNA was extracted and purified from the serum samples using the QIAamp Viral Mini Extraction kit (Qiagen, Hilden, Germany), following the kit manufacturer’s instructions. The quantity and the purity of the extracted RNA were verified by spectroscopy (NanoDrop 1000, Thermo Scientific, USA).

Genesig multiplex kit (Primerdesign Ltd, UK) was used to test for the presence or otherwise of chikungunya, dengue, and zika viruses’ RNA in a single reaction [21]. The kit contained Osig One-step master mix, multiplex primer/probe mix, and RNase/DNase free water. After reconstituting the various components of the test kits following the manufacturer’s instructions, a multiplex RT-qPCR was performed in a 20 μl reaction volume that comprised 10 μl of Osig One-step master mix, 1 μl multiplex primer/probe mix, 4 μl of RNase/DNase free water and 5 μl of the RNA sample. Amplification was performed using the CFX96 Touch Real-Time PCR Detection System (Bio-Rad, USA). The RT-qPCR cycling conditions were as follows: 55°C for 10 minutes for one cycle to allow for cDNA synthesis; 95°C for 2 minutes to allow for activation of Tag polymerase followed by 95°C for 10 seconds for 50 cycles (denaturation) and 60°C for 60 seconds for 50 cycles (annealing/extension). The RT-qPCR reaction products were analysed with Bio-Rad CFX 96 software (S1 Fig). A single positive control that contained RNA templates for each of the three viruses was used, while the negative control was RNase-free water; both supplied by the kit manufacturer. The details of primers and probes sequences were not provided by the manufacturer and therefore cannot be cited.

(https://www.genesig.com/assets/files/dengue_chikv_zikv_multiplex_handbook.pdf)

### Statistical analysis

The data generated were analysed with IBM Statistical Package for Social Sciences (SPSS) version 20 (IBM Corporation, USA). Continuous data were expressed as mean ± standard deviation (SD). Categorical variables were expressed in percentages and frequencies, and Chi-square (χ^2^) test was used to determine significant differences between categorical variables, while Fisher exact test was used in cases when expected counts were less than 5. A *p-*value of less than 0.05 was considered statistically significant.

## Results

### Chikungunya seroprevalence and malaria status of study participants

Data on 600 participants comprising 226 (37.7%) males and 374 (62.3%) females were analysed in this study. More females than males participated in the study; 63% (126/200) were from the Holy Family Hospital, 62.5% (125/200) from the Sunyani Municipal Hospital, and 61.5% (123/200) from Manhyia District Hospital. The mean age of the participants was 26.5 years (±20.1) and the ages ranged between 1 and 91 years. The majority of the participants were less than 11 years (29%), and most were from the Holy Family and Sunyani Municipal Hospitals with 41.5% and 40.0% respectively, with the least (5.5%) from the Manhyia District Hospital. The age group with the least participants was the above-60-years group representing 5.8% of the total study participants; Manhyia District Hospital had the highest proportion (7.0%), followed by Sunyani Municipal Hospital (6.5%), and the least was Holy Family Hospital (4.0%).

Of the 600 study participants enrolled in the study, the overall CHIKV antibodies (IgM and IgG) detected was 6% (36/600). The seroprevalences for CHIKV IgM and IgG antibodies were 1.8% (11/600) and 4.2% (25/600) respectively; however, 8.3% (3/36) of the overall seroprevalence (representing 0.5% of 600) had both IgM and IgG antibodies. The IgM (4.5%) and IgG (12%) seropositivities were both highest at the Manhyia District Hospital. There was a significant association between CHIKV IgM antibodies and the sites of sampling (*p*-value = 0.003), but not between IgG and the sampling sites (*p-*value = 0.274). The prevalence of malaria detected in the current study was 32.3% (194/600). Malaria prevalence was higher (46%) at Manhyia District Hospital and the distribution of malaria positivity at the hospitals was significant, (*p* < 0.001) (Table 1).

**Table 1:** Chikungunya seroprevalence and malaria status of study participants.

	*n*	IgM (%)	*p*-value	IgG (%)	*p*-value	IgM & IgG (%)	*p*-value	Malaria (%)	*p*-value
*Overall*	600	11 (1.8)		25 (4.2)		3 (0.5)		194 (32.3)	
*Sex*									
Male	226	7 (63.6)	^b^0.112	6 (24.0)	^a^0.149	1 (33.3)	^b^1.000	81 (41.8)	^a^0.153
Female	374	4 (36.4)		19 (76.0)		2 (66.7)		113 (58.2)	
*Age*									
< 11	174	0	^b^0.57	1 (4.0)	^b^0.002	0	^b^0.27	62 (32.0)	^a^0.327
11–20	94	1 (9.1)		3 (12.0)		0		35 (18.0)	
21–30	104	4 (36.4)		4 (16.0)		2 (66.7)		29 (14.9)	
31–40	69	3 (27.3)		6 (24.0)		0		15 (7.7)	
41–50	73	2 (18.2)		4 (16.0)		1 (33.3)		25 (12.9)	
51–60	51	1 (9.1)		2 (8.0)		0		18 (9.3)	
> 60	35	0		5 (20.0)		0		10 (5.2)	
*Hospital*									
Holy Family	200	1 (0.5)	^b^0.004	7 (3.5)	^a^0.274	0 (0.0)	^b^0.11	41 (20.5)	^a^<0.001
Manhyia District	200	9 (4.5)		12 (6.0)		3 (1.5)		92 (46.0)	
Sunyani Municipal	200	1 (0.5)		6 (3.0)		0 (0.0)		61 (30.5)	

^**a**^ **Difference calculated by Chi-square test**

^**b**^ **Difference calculated by Fisher exact test**

Male participants were observed to have higher CHIKV IgM seropositivity of 63.3%. On the other hand, female participants were observed to have higher (76%) CHIKV IgG seropositives and 58.2% malaria positives. A Chi-square test did not reveal statistically reliable differences between gender and with the various CHIKV IgM and IgG antibodies and malaria positivity (*p*-values = 0.112, 0.149, and 0.153 respectively) (Table 1).

Participants within the age group of 21–30 years were observed to have a higher proportion (36.4%) of CHIKV IgM seropositives. Those within the age group of 31–40 years had a higher proportion (24%) of CHIKV IgG positives and a Chi-square test did reveal a statistically significant difference between IgG and the age groups (*p* = 0.005). A larger proportion (35%) of malaria positives were detected among participants within the age group of 11–20 years (Table 1). Of the 11 participants who tested positive for CHIKV IgM, 36% (4/11) were positive for malaria. Also, 28% (7/25) of the CHIKV IgG positive samples tested positive for malaria. Three participants of the overall seroprevalence had both CHIKV IgM and IgG antibodies detected concurrently and of this, two (66.7%) also tested positive for malaria parasites. There were no statistical differences in the distributions of CHIKV antibodies and malaria positivity.

### Clinical presentation of the study participants

The most common symptoms reported among the patients were headache (57.9%), muscular pains (46.0%), chill, shivering & sweating (42.2%), joint pain (41.7%), nausea/vomiting (16.5%), and rashes (7.3%). Most of the patients’ positive for IgM (2.0%), IgG (5.5%), and malaria (34.2%) presented with headaches, however, no statistical differences were found in these distributions (Table 2). Lower proportions of IgM (2.9%) and IgG (33.3%) positive patients reported muscular pains, though no statistical associations were observed (*p* = 0.07 and 0.41, respectively). Lower proportions of patients with the reported symptoms were positive for CHIKV antibodies and malaria parasites; 0.4% for joint pains, 1.8% for muscular pains, 2.0% for chills, 2.0% for headache, and 3.0% for nausea and vomiting. No significant differences were observed among patients positive for both CHIKV antibodies plus malaria parasites and reported symptoms (Table 2).

**Table 2:** Association of symptoms with chikungunya IgM, IgG, and Malaria.

		CHIK IgM			CHIK IgG			CHIK + Malaria		
Symptom	*n*	Positive (%)	Negative (%)	*p*-value	Positive (%)	Negative (%)	*p*-value	Positive (%)	Negative (%)	*p*-value
Joint pains										
Yes	250	3 (1.2)	247 (98.8)	^b^0.37	11 (4.4)	239 (95.6)	^a^0.82	1 (0.4)	(99.6)	^b^0.09
No	349	8 (2.3)	341 (97.7)		14 (4.0)	335 (96.0)		8 (2.3)	341 (97.7)	
Muscular pains										
Yes	273	8 (2.9)	265 (97.1)	^a^0.07	13 (33.3)	26 (66.7)	^a^0.41	5 (1.8)	268 (98.2)	^b^0.07
No	321	3 (0.9)	318 (99.1)		11 (3.4)	310 (96.6)		4 (1.2)	317 (98.8)	
Rashes										
Yes	44	1 (2.3)	43 (97.7)	^b^0.57	2 (4.5)	42 (95.5)	^b^0.70	0	44 (100.0)	^b^1.00
No	556	10 (1.8)	546 (98.2)		23 (4.1)	533 (95.9)		9 (1.6)	547 (98.4)	
Chills/Shivering/Sweating										
Yes	248	6 (2.4)	242 (97.6)	^b^0.54	12 (4.8)	236 (95.2)	^a^0.43	5 (2.0)	243 (98.0)	^b^0.50
No	340	5 (1.5)	335 (98.5)		12 (3.5)	328 (96.5)		4 (1.2)	336 (98.8)	
Headache										
Yes	345	7 (2.0)	338 (98.0)	^b^0.77	19 (5.5)	326 (94.5)	^a^0.06	7 (2.0)	338 (98.0)	^b^0.31
No	251	4 (1.6)	247 (98.4)		6 (2.4)	245 (97.6)		2 (0.8)	249 (99.2)	
Nausea/Vomiting										
Yes	99	4 (4.0)	95 (96.0)	^b^0.09	4 (4.0)	95 (96.0)	^b^1.00	3 (3.0)	96 (97.0)	^b^0.17
No	501	7 (1.4)	495 (98.6)		21 (4.2)	478 (95.8)		6 (1.2)	493 (98.4)	

^**a**^ **Difference calculated by Chi-square test**

^**b**^ **Difference calculated by Fisher exact test**

## Discussion

The current study has provided evidence of CHIKV exposure among the study participants in the Ashanti, Bono, and Bono East Regions of Ghana. The overall CHIKV specific antibodies (IgM and IgG) seropositivity was 6%. IgM and IgG seropositivities were 1.8% and 4.2% respectively. The overall CHIKV specific seropositivity reported in the current study was lower than the 27.69% reported by Manu *et al*., (2019) in Accra Ghana [22], 50.17% by Baba *et al*., (2013) [23], 41.8% by Olajiga *et al*., (2017) [19] and 24.7% reported by Udeze *et al*., (2019) [24] in separate and independent studies conducted in different parts of Nigeria. However, the overall low CHIKV antibodies seropositivity observed in the current study was comparable to that (4.1%) reported by Akinola *et al*., (2017) in Nigeria [25]. The low CHIKV specific IgM seropositivity detected in the current is consistent with that reported in a study in Tanzania by Kinimi *et al*., (2018) [26]. The detection of IgM antibodies could point to recent infections and that the virus may be in circulation among the population. Studies by Sissoko *et al*., (2008) [27], Azami *et al*., (2013) [28], and Olajiga *et al*., (2017) [19] reported significant associations between anti-chikungunya virus antibodies distribution and gender. However, no significant difference in anti-chikungunya antibodies distribution between the genders was observed in this current study. IgM seropositive was higher in males than in females in the current study. This could be attributed to the higher likelihood that males are generally more likely to spend more time outdoors than females, and hence are more likely to be exposed to the vectors than females.

Conversely, IgG seropositivity was higher in females and was similar to that reported by Olajiga *et al*., (2017) [19] but differs from that reported by Kinimi *et al*., (2018) [26]. Seroprevalence was higher in adults, as they form the most active group in going to farms and forests where the likelihood of being bitten by mosquitoes is very high. In the urban setting, these adults tend to spend long periods outside as part of their lifestyles, making them susceptible to bites from the *Aedes* mosquitoes which prefer daytime and dusk to feed [29,30]. Notwithstanding, the reported cases of malaria in this study stood at (32.3%) and was higher among females. This finding is inconsistent with that reported by Kinimi *et al*., (2018) [26].

Chikungunya-malaria co-infection, however, was lower at 1.8% (11/600) in this study. This low co-infection rate observed was similar to studies done in different areas across sub-Sahara Africa reported as; 6.7% by Baba *et al*., (2013) [23], 0.54% by Chipwaza *et al*., (2014) [31], 15% by Ayorinde *et al*., (2016) [32], 0.02% by Sow *et al*., (2016) [30], 7.14% by Kinimi *et al*., (2018) [26] and 1.23% by Mugabe *et al*., (2018) [33]. However, the low prevalence of chikungunya virus exposure explains the low prevalence of co-infection with malaria observed. The low prevalence of chikungunya-malaria co-infections could also be attributed to different transmission vectors for malaria parasites and the chikungunya virus [34]. The *Anopheles* mosquitoes, vectors of the malaria parasite, is a night-biting mosquito while the *Aedes* mosquitoes, vectors of chikungunya virus, is daytime feeder [35]. Studies from Ghana showed the *Aedes* mosquitoes are actively circulating and could potentially promote outbreaks of viral haemorrhagic fevers [13,14]. The presence of these actively circulating *Aedes* mosquitoes could potentially be the source of the low-level circulating chikungunya virus identified in this study.

With no statistical associations, the most commonly reported clinical symptoms among the chikungunya and malaria infections aside from the febrile condition were headache, muscular pains, chills, shivering & sweating, joint pains, and nausea/vomiting. This result is in line with that of other studies [22,26,29,31]. The current study did not record any positive signal for either chikungunya virus, dengue virus, or Zika virus RNA detection by RT-qPCR–a similar observation was reported here in Ghana by Manu *et al*., [22]. Dash *et al*., (2011) pointed out that the detection of chikungunya viral RNA is from the day of infection or onset of symptoms to day 7, after which acute infections detection is limited [36]. The non-detection of viral RNA could be due to the timing of the blood sampling which might have occurred after the acute phase since most Ghanaians self-medicate first before seeking proper medical attention at the hospitals [37]. It is recommended that the study be conducted on a large scale nationwide to strengthen the data.

### Limitation of the study

The details of primers and probes sequences were not provided by the manufacturer although the authors requested for the details. Moreover, the RDT test used for malaria detection among the study participants is of lower sensitivity compared to the PCR and so inference based on CHIKV-malaria co-infection should be made with caution.

## Conclusion

This study report evidence of chikungunya virus exposure among Ghanaians in the study areas. The detection of chikungunya antibodies or exposure points to an unnoticed transmission intensity of the virus among patients who patronised health services at the health facility. The true burden of the virus and its associated disease is still not known though the country is endemic with the vector implicated in the transmission of this virus. It is anticipated that these findings would provoke a wider discussion about the burden of chikungunya virus infections. This will help guide policymakers to develop effective and affordable early warning and outbreak response systems for Ghana.

## Supporting information

S1 Questionnaire Questionnaire on ‘Molecular and Serological Evidence of Chikungunya virus infection among suspected malaria cases at Manhyia District Hospital, Kumasi–Ashanti, Sunyani Municipal Hospital, Sunyani–Bono and Holy Family Hospital, Techiman–Bono East. (DOCX)

S1 Fig Amplification and detection of viral nucleic acids. Chikungunya, Dengue and Zika viruses RNAs were amplified and detected with Genesig Real Time PCR detection kit using CFX 96 Touch Real-Time PCR detection system. The chikungunya virus RNA was detected using CY5 fluorescent channel while Dengue and Zika viral RNAs were detected using VIC and FAM fluorescent channels respectively. All the samples tested were below the RNA detection threshold. However, the positive controls for the three viruses tested came out positive. The pink curve represent positive control for chikungunya virus while the green and blue curves represent the positive controls for Zika and Dengue viruses respectively.
(TIF)
