# Supplementary material for: Evidence of chikungunya virus infections among febrile patients at three secondary health facilities in the Ashanti and the Bono Regions of Ghana
Source: PLoS Negl Trop Dis. 2021 Aug 30;15(8):e0009735. doi: 10.1371/journal.pntd.0009735 (PMC8432890; doi:10.1371/journal.pntd.0009735)
Supplement: S1 Questionnaire — (DOCX) [file pntd.0009735.s001.docx]

# KWAME NKRUMAH UNIVERSITY OF SCIENCE AND TECHNOLOGY

**COLLEGE OF HEALTH SCIENCES**

**SCHOOL OF MEDICAL SCIENCES**

**DEPARTMENT OF CLINICAL MICROBIOLOGY**

# To Whom It May Concern:

# Dear Respondent,

# Questionnaire on ‘Molecular and Serological Evidence of Chikungunya virus infection among suspected malaria cases at Manhyia District Hospital, Kumasi – Ashanti, Sunyani Municipal Hospital, Sunyani – Bono and Holy Family Hospital, Techiman – Bono East.

*Our research team is conducting a study on the above topic. We kindly ask you to answer this questionnaire as part of our study and academic exercise. The study will involve anyone who has been presumptively diagnosed as having malaria and asked to conduct a laboratory test for confirmation.*

***We guarantee that information provided would be kept confidential as the names of participants will be coded.***

***Hospital***: **Manhyia District Hospital [ ] Sunyani Municipal Hospital [ ] Holy Family Hospital [ ]**

Case identification number: ……….………………….… Date: …............................................

Name: …………………………….….……………………………....…………………………

Path No: ……………………. Serial Number: ….…………… O.P.D Number: ……………...

**SOCIO-DEMOGRAPHICS OF STUDY PARTICIPANTS**

1. Age: ……………..
2. Sex A. Male [ ] B. Female [ ]
3. Level of education A. No formal education [ ] B. Kindergarten [ ] C. Primary [ ]

D. J.H.S. [ ] E. S.H.S. [ ] F. Other(s), specify……………

**CLINCAL INFORMATION OF THE STUDY PARTICIPANTS**

1. Arthritis at the A. Joints □ B. Ankle □ C. Wrist □ D. Fingers □ E. Toes □
2. Myalgia (Muscle pain) A. Yes □ B. No □
3. Body temperature …… A. (36 – 37°C) □ B. (37.1 – 38.5°C) □ C. Fever (>38.5°C) □
4. Rashes on A. Ankle □ B. Feet □ C. Other body parts □ D. All over the body □
5. Chills, Shivering and Sweating A. Yes □ B. No □
6. Headache A. Yes □ B. No □
7. Nausea or vomiting A. Yes □ B. No □
8. Onset of symptoms A. 1 – 3 days □ B. 4 – 6 days □ C. > 7days □ D. Unknown □
9. Where do you live (Town/Region)? ……………………………………………..…
10. Do you sleep under mosquito net? A. Yes □ B. No □
11. Have you travel outside Ghana within the past month? A. Yes □ B. No □
12. If yes (to Q14 above), to which country? ………………………………..………
13. Results of Malaria test A. Positive [ ] B. Negative [ ]
