# Supplementary figures and images for: Evidence of chikungunya virus infections among febrile patients at three secondary health facilities in the Ashanti and the Bono Regions of Ghana
Source: PLoS Negl Trop Dis. 2021 Aug 30;15(8):e0009735. doi: 10.1371/journal.pntd.0009735 (PMC8432890; doi:10.1371/journal.pntd.0009735)

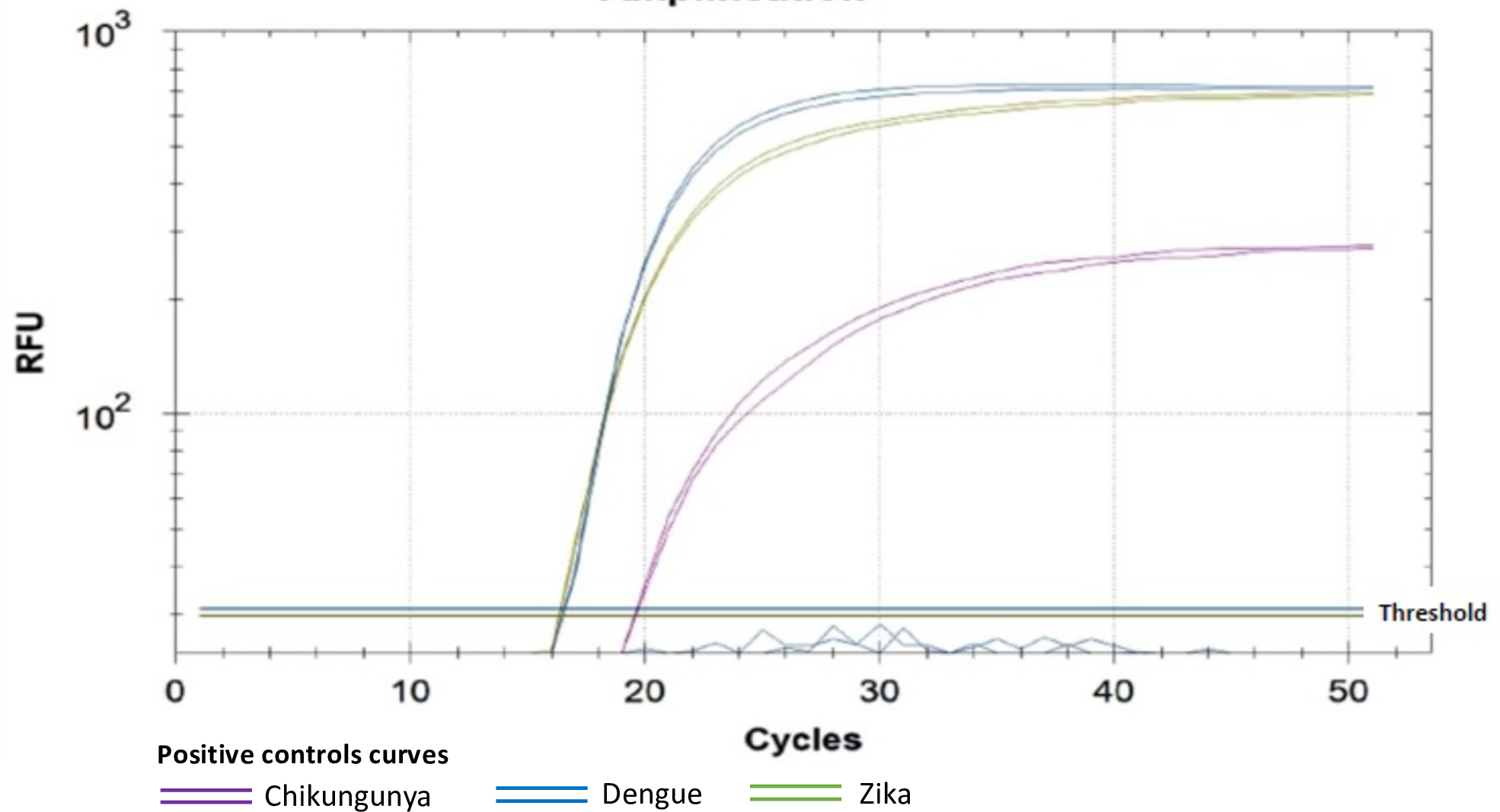

Supplement: S1 Fig — Chikungunya, Dengue and Zika viruses RNAs were amplified and detected with Genesig Real Time PCR detection kit using CFX 96 Touch Real-Time PCR detection system. The chikungunya virus RNA was detected using CY5 fluorescent channel while Dengue and Zika viral RNAs were detected using VIC and FAM fluorescent channels respectively. All the samples tested were below the RNA detection threshold. However, the positive controls for the three viruses tested came out positive. The pink curve represent positive control for chikungunya virus while the green and blue curves represent the positive controls for Zika and Dengue viruses respectively. (TIF) [file pntd.0009735.s002.tif]
